# Soy Isoflavone Genistein Enhances Tamoxifen Sensitivity in Breast Cancer via microRNA and Glucose Metabolism Modulation

**DOI:** 10.3390/ijms26020733

**Published:** 2025-01-16

**Authors:** Jessica Shpigel, Emilia F. Luciano, Blessing Ukandu, Moira Sauane, Columba de la Parra

**Affiliations:** 1 Department of Chemistry, Herbert H. Lehman College, City University of New York, New York, NY 10468, USA; jessica.shpigel@lc.cuny.edu (J.S.); emilia.carlo@lc.cuny.edu (E.F.L.); blessing.ukandu@lc.cuny.edu (B.U.); 2 Department of Biological Sciences, Herbert H. Lehman College, City University of New York, New York, NY 10468, USA; moira.sauane@lehman.cuny.edu; 3 Biochemistry, Biology, and Chemistry Ph.D. Programs, The Graduate Center, City University of New York, New York, NY 10016, USA

**Keywords:** breast cancer, isoflavone, genistein, natural compounds, microRNA, glucose metabolism, tamoxifen

## Abstract

Breast cancer treatment has advanced significantly, particularly for estrogen receptor-positive (ER+) tumors. Tamoxifen, an estrogen antagonist, is widely used; however, approximately 40% of patients develop resistance. Recent studies indicate that microRNAs, especially miR-155, play a critical role in this resistance. Our analysis of MCF-7 tamoxifen-sensitive (TAM-S) and tamoxifen-resistant (TAM-R) cells revealed that miR-155 is significantly upregulated in TAM-R cells. Overexpression of miR-155 in TAM-S cells increased resistance to tamoxifen. Additionally, genistein, a natural isoflavone from soybeans, effectively downregulated miR-155 and its targets associated with apoptosis and glucose metabolism, including STAT3 and hexokinase 2 (HK2). Notably, genistein also significantly decreased cell migration, suggesting potential anti-metastatic effects. Furthermore, genistein reduced glucose consumption, indicating its potential to overcome miR-155-mediated tamoxifen resistance and modulate the Warburg effect. These findings highlight genistein as a promising therapeutic agent for overcoming tamoxifen resistance in ER+ breast cancer and merit further investigation.

## 1. Introduction

Breast cancer is the most frequently diagnosed cancer among women worldwide, with approximately 70% of cases being estrogen receptor-positive (ER+) [1,2]. Tamoxifen, a selective estrogen receptor modulator (SERM), has long been a foundation of treatment for ER+ breast cancer. However, around 40% of patients develop resistance to tamoxifen, representing a significant challenge in cancer therapy [3,4].

Advances in cancer research have highlighted the essential role of microRNAs (miRNAs) in regulating gene expression and contributing to drug resistance. MiRNAs are small, non-coding RNAs (18–25 nucleotides) that modulate gene expression post-transcriptionally by targeting messenger RNAs (mRNAs) for degradation or translational repression. Dysregulated miRNA expression has been implicated in various cancers, affecting critical processes such as cell proliferation, apoptosis, metabolism and metastasis [5,6]. Among these, miR-155 has emerged as a key regulator in breast cancer progression and therapy resistance. Overexpression of miR-155 is associated with poor prognosis in several cancers, including breast and lung cancers [7]. In breast cancer, elevated miR-155 levels have been observed in tamoxifen-resistant tumors, suggesting its involvement in the molecular mechanisms underlying tamoxifen resistance [8,9]. Specifically, miR-155 contributes to tamoxifen resistance by targeting the suppressor of cytokine signaling 6 (SOCS6), which normally functions as a negative feedback regulator of the Janus kinase/signal transducer and activator of transcription (JAK/STAT) pathway. By inhibiting SOCS6, miR-155 enhances signal transducer and activator of transcription 3 (STAT3) signaling, promoting cell survival and proliferation in tamoxifen-resistant cells [10].

There is a complex interplay of miRNAs in therapeutic responses. Studies have shown that microRNAs, such as miR-486-5p, can synergize with tamoxifen to induce profound cell death in tamoxifen-resistant breast cancer cells [10]. Furthermore, long non-coding RNA MIR497HG and its derived microRNAs, miR-195 and miR-497, mediate tamoxifen resistance through the phosphoinositide 3-kinase (PI3K)/protein kinase B (AKT) signaling pathway [11]. Additionally, the miR-186-3p/EREG axis has been identified as a critical regulator of aerobic glycolysis in tamoxifen-resistant breast cancer cells, promoting resistance by enhancing glycolytic pathways [12].

Recent studies have demonstrated that altered glucose metabolism significantly contributes to tamoxifen resistance in breast cancer. Enhanced glycolysis, known as the Warburg effect, is a hallmark of cancer cells and has been linked to various drug resistance mechanisms. This metabolic shift provides cancer cells with the necessary energy and substrates for rapid growth and survival [1,13,14]. High glucose levels can impair tamoxifen responsiveness by modulating connective tissue growth factor (CTGF) levels in breast cancer cells. Elevated expression of hexokinase II (HK2) has been associated with increased glycolytic activity and resistance to tamoxifen. Notably, HK2 was shown to inhibit the mechanistic target of rapamycin (mTOR)–S6 kinase (S6K) signaling pathway. This inhibition led to enhanced autophagy, which conferred resistance in ER+ cancer cells [15]. Research indicates that shifting from high glucose to low glucose conditions can improve sensitivity to tamoxifen, highlighting the impact of glucose levels on drug efficacy [16]. The Warburg effect not only supports cancer cell proliferation but also contributes to a tumor microenvironment that fosters drug resistance, suggesting that targeting these metabolic pathways may enhance treatment outcomes for patients with estrogen receptor-positive breast cancer [14,17,18].

Genistein (4′,5,7-trihydroxyisoflavone), an isoflavone predominantly found in soybeans, has been extensively studied for its anticancer properties in both preclinical and clinical settings [19,20,21,22]. Genistein exerts its anticancer effects through multiple mechanisms—both estrogen-dependent and independent—by inhibiting cancer cell proliferation, inducing apoptosis, suppressing metastasis and modulating miRNA expression [13,14]. Notably, it downregulates oncogenic miRNAs such as miR-155, which is upregulated in cancers [23,24,25]. Genistein achieves this modulation by targeting several cellular signaling pathways associated with cell cycle regulation and apoptosis, particularly the PI3-K/Akt pathway. The downregulation of miR-155 correlates with increased expression of proapoptotic factors, such as FOXO3 [23]. Importantly, genistein’s ability to alter glucose metabolism may significantly contribute to its potential in overcoming tamoxifen resistance. Recent studies have shown that genistein can inhibit glucose uptake in cancer cells by downregulating glucose transporter 1 (GLUT1) and key glycolytic enzymes [26]. This metabolic modulation may help reverse the glycolytic phenotype associated with tamoxifen-resistant tumors, potentially resensitizing them to treatment. Furthermore, genistein has demonstrated synergistic effects when combined with other anticancer agents, enhancing their therapeutic efficacy and positioning it as a promising addition to existing cancer treatments [27].

This study aims to investigate the role of miR-155 in tamoxifen-resistant breast cancer cells and evaluate the potential of genistein to modulate miR-155 expression and restore sensitivity to tamoxifen. Our findings provide valuable insights into the molecular mechanisms contributing to tamoxifen resistance, particularly through the modulation of microRNAs and glucose metabolism, highlighting genistein as a potential therapeutic agent. These results contribute to the growing evidence supporting genistein’s therapeutic potential as a strategy to overcome tamoxifen resistance and improve treatment outcomes for patients with ER+ breast cancer.

## 2. Results

### 2.1. miR-155 Expression in Tamoxifen-Resistant Cells

**Figure 1:**
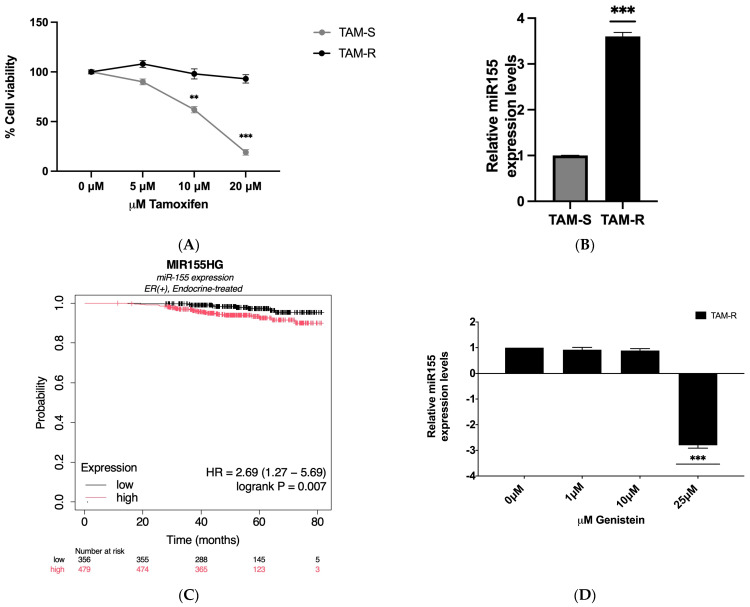
Effects of tamoxifen, miR-155 and genistein on MCF7 tamoxifen-sensitive (TAM-S) and tamoxifen-resistant (TAM-R) cells. (**A**) Cell viability of TAM-S and TAM-R cells following tamoxifen treatment, measured by the MTT assay. (**B**) Relative expression levels of miR-155 in TAM-S and TAM-R cells, quantified by RT-qPCR. (**C**) Kaplan–Meier analysis of relapse-free survival in ER-positive breast cancer patients treated with endocrine therapy, showing a significant difference in survival between high and low MIR155HG expression groups (HR = 2.69, 95% CI: 1.27–5.69; log-rank *p* = 0.007). (**D**) Changes in miR-155 expression in TAM-R MCF7 cells after genistein treatment, measured by RT-qPCR. Data are presented as mean ± SEM (*n* = 3). Statistical significance was set at *p* < 0.05. Significant differences relative to the control are indicated by asterisks: ** *p* < 0.01, *** *p* < 0.001.

MCF7 tamoxifen-sensitive (TAM-S) cells and their matched tamoxifen-resistant (TAM-R) counterparts were used to investigate resistance to tamoxifen therapy [28]. To validate the model, we assessed the sensitivity of TAM-S and TAM-R cells to tamoxifen treatment by exposing them to increasing concentrations (0–20 μM) for 24 h, measuring cell viability with the MTT assay. As shown in Figure 1A, TAM-S cells exhibited a dose-dependent decrease in cell viability, while TAM-R cells demonstrated significant resistance, with less than 10% reduction in viability across all concentrations. These results confirm TAM-R cells as a model for studying tamoxifen resistance.

MicroRNAs (miRNAs) play a crucial role in regulating gene expression and contributing to drug resistance in various cancers. Among these, miR-155 has emerged as a key player in breast cancer progression and therapy resistance [9,23]. Given that tamoxifen-resistant cells exhibit a more aggressive phenotype than their tamoxifen-sensitive counterparts, we investigated the role of this oncomiR in the tamoxifen resistance phenomenon. To elucidate the role of miR-155 in tamoxifen resistance, we first analyzed its expression in tamoxifen-resistant (TAM-R) and tamoxifen-sensitive (TAM-S) cells using RT-qPCR. As shown in Figure 1B, miR-155 was significantly upregulated in TAM-R cells, showing more than a three-fold increase compared to TAM-S cells. This upregulation aligns with previous findings that associate elevated miR-155 levels with poor prognosis in breast cancer [9].

To further validate the clinical significance of miR-155 in tamoxifen resistance, we performed a Kaplan–Meier plotter analysis [29]. The results indicated that increased expression of miR-155 is significantly associated (*p* = 0.007) with poor survival in patients with estrogen receptor (ER)-positive breast cancer who received endocrine treatment (Figure 1C). The analysis reveals a significant difference in relapse-free survival between groups, with a hazard ratio (HR) of 2.69 (95% CI: 1.27–5.69), indicating that high MIR155HG expression is associated with a nearly three-fold increase in the risk of relapse. This finding highlights the critical role of miR-155 in patient outcomes and its potential as a prognostic marker. These results are consistent with studies in the literature that have reported a significant association between high miR-155 expression and lower disease-free survival and overall survival rates in breast cancer patients. Furthermore, miR-155 has been suggested as an independent prognostic factor and a potential therapeutic target [30,31].

### 2.2. miR-155 Modulation by Genistein and Its Effect on Tamoxifen Resistance

Given the established anticancer properties of genistein and its ability to modulate microRNA expression, particularly its effect on miR-155 in aggressive breast cancers [23,32], we investigated its potential in tamoxifen-resistant cells. Considering the increased aggressiveness and invasiveness of tamoxifen-resistant cells compared to their tamoxifen-sensitive counterparts, we hypothesized that genistein might have similar effects on miR-155 expression in these cells. TAM-R MCF-7 cells were treated with various concentrations of genistein (0, 1, 10 and 25 µM), selected based on previous studies demonstrating their efficacy in inhibiting cancer cell growth [19,20]. To assess the impact of genistein on miR-155 expression, we performed RT-qPCR analysis. The results indicated that genistein treatment significantly reduced miR-155 expression in TAM-R cells. Notably, at the 25 µM concentration, genistein reduced miR-155 levels by three-fold compared to the control group (Figure 1D). However, intermediate concentrations of genistein (1 µM and 10 µM) did not produce statistically significant changes in miR-155 expression. Since 25 μM genistein was the most effective concentration for reducing miR-155 levels, and this concentration has also been shown to significantly decrease cancer cell growth and act synergistically with other anticancer agents, subsequent experiments were conducted at this concentration [27,33,34]. These findings suggest that genistein can effectively modulate miR-155 expression in tamoxifen-resistant cells.

### 2.3. miR-155 Targets and Glucose Metabolism

MicroRNAs have emerged as key regulators of metabolic processes, which are the hallmark features of cancer [35,36]. Enhanced glycolysis and the Warburg effect are central to cancer metabolism, particularly in aggressive cancer phenotypes, and have been shown to contribute to mechanisms of endocrine resistance [37]. STAT3, a key metabolic regulator and transcriptional activator of miR-155 [38], plays a pivotal role in glucose metabolism. Notably, overexpression of miR-155 has been associated with increased phosphorylation of STAT3 [38,39], suggesting a complex regulatory loop. Moreover, hexokinase 2 (HK2), a rate-limiting enzyme in glycolysis, a transcriptional target of STAT3 and crucial for the Warburg effect, contributing to altered glucose metabolism in cancer cells, has been implicated in tamoxifen resistance [40].

To explore these interactions further, the effects of genistein on the expression of STAT3, phosphorylated STAT3 (p-STAT3) and HK2 were assessed using immunoblot analysis. The immunoblot analysis revealed distinct effects of genistein treatment on key proteins involved in tamoxifen resistance and glucose metabolism. Total STAT3 expression remained unchanged in both TAM-R and TAM-S cells, regardless of treatment. However, phosphorylation of STAT3, a marker of its activation, was significantly higher in TAM-R cells compared to TAM-S cells, and this phosphorylation was notably reduced following genistein treatment. Similarly, the expression of HK2, a key enzyme in glycolysis, was also reduced after genistein treatment. This decrease is particularly significant, as HK2 is a rate-limiting enzyme in glycolysis, and its upregulation is often associated with aggressive cancers. HK2 has been shown to be upregulated in aggressive cancers, and our results confirm its increased expression in TAM-R cells relative to TAM-S cells. STAT3 plays a crucial role in advanced cancers by promoting glycolysis through the transcriptional activation of HIF-1α and HK2 in vitro [1]. Both STAT3 and HK2 are critical components of the Warburg effect, contributing to altered glucose metabolism in cancer cells.

Given this significance, we evaluated the impact of genistein on glycolysis in both tamoxifen-sensitive (TAM-S) and tamoxifen-resistant (TAM-R) breast cancer cell models. We measured glucose consumption, a key marker of anaerobic glycolysis and a central feature of the Warburg effect, to assess glycolytic activity.

**Figure 2:**
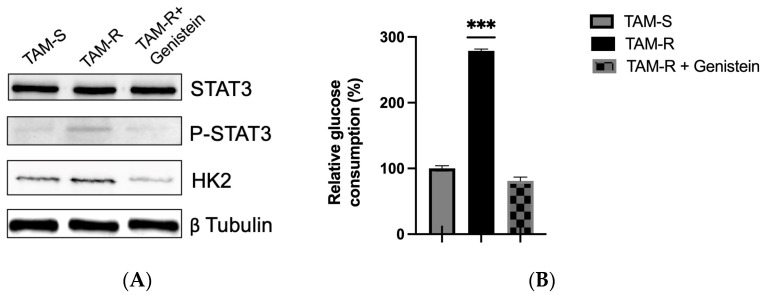
Analysis of STAT3, p-STAT3, HK2 expression and glucose consumption in tamoxifen-sensitive and tamoxifen-resistant MCF7 cells. (**A**) Representative immunoblot showing the protein levels of STAT3, p-STAT3, HK2 and β-tubulin in TAM-S and TAM-R MCF7 cells, with and without genistein treatment. (**B**) Relative glucose consumption in TAM-S and TAM-R MCF7 cells, with and without genistein treatment. Data are presented as mean ± SEM (*n* = 3). Statistical significance was set at *p* < 0.05. Significant differences relative to the control are indicated by asterisks: *** *p* < 0.001.

Our results showed that glycolysis was significantly upregulated in tamoxifen-resistant (TAM-R) cells compared to tamoxifen-sensitive (TAM-S) cells. However, treatment with genistein resulted in a marked reduction in glycolytic activity in TAM-R cells, bringing it closer to the levels observed in TAM-S cells (Figure 2B). These findings suggest that genistein may partially restore normal glycolytic activity in TAM-R cells, potentially reversing resistance to tamoxifen. The ability of genistein to modulate glycolytic activity in TAM-R cells to levels comparable with those seen in tamoxifen-sensitive cells indicates its potential as a therapeutic agent in overcoming tamoxifen resistance in breast cancer.

The decrease in glycolysis observed in genistein-treated tamoxifen-resistant (TAM-R) breast cancer cells is likely linked to important changes in HK2 and STAT3 expression. STAT3, a transcription factor, activates multiple genes involved in glycolysis, including HK2, a rate-limiting enzyme that promotes glucose phosphorylation and enhances glycolytic activity. Our results show that genistein reduces STAT3 phosphorylation in TAM-R cells, leading to lower HK2 expression and a subsequent decrease in glycolytic rate. This is consistent with genistein’s known ability to inhibit aerobic glycolysis in cancer cells.

Additionally, STAT3 regulates the activity of various glycolytic enzymes and glucose transporters, including GLUT1 [1]. Thus, its inhibition by genistein could impact multiple steps in the glycolytic pathway. Importantly, STAT3 is also a transcriptional activator of miR-155, which we found to be downregulated by genistein treatment [38]. This suggests a complex regulatory network involving STAT3, miR-155 and glycolytic enzymes in maintaining the tamoxifen-resistant phenotype.

### 2.4. Effect of Genistein on Cell Migration and Cell Viability

**Figure 3:**
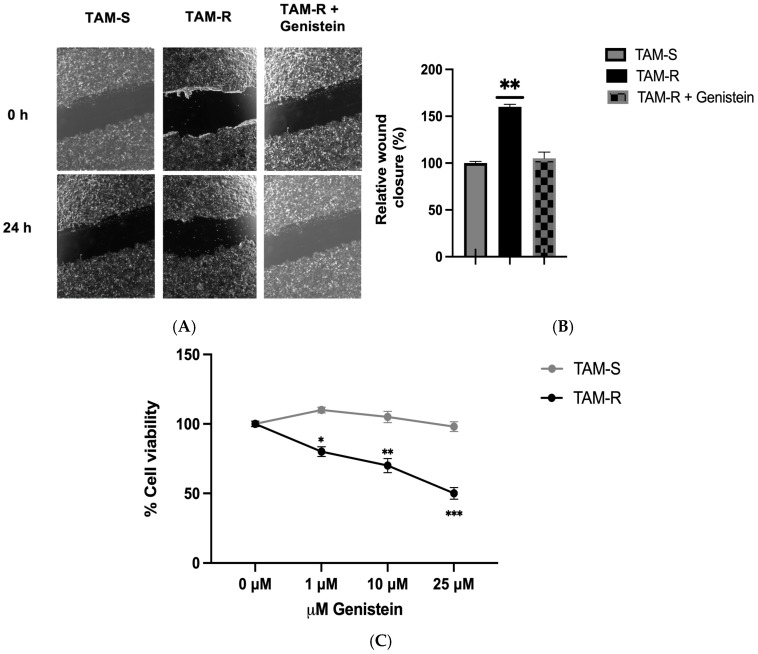
Effect of genistein on cell migration and cell viability in TAM-S and TAM-R MCF7 cells. (**A**) Representative images of cell migration (wound healing) assays in TAM-S and TAM-R MCF7 cells, with or without 25 µM genistein treatment for 24 h, captured using a digital inverted microscope (40× magnification). (**B**) Quantification of relative wound closure in TAM-S and TAM-R MCF7 cells treated with or without 25 µM genistein for 24 h. (**C**) Cell viability measured by the MTT assay in TAM-S and TAM-R MCF7 cells, with or without 25 µM genistein treatment. Data are presented as mean ± SEM (*n* = 3). Statistical significance was set at *p* < 0.05. Significant differences relative to the control are indicated by asterisks: * *p* < 0.05, ** *p* < 0.01, *** *p* < 0.001.

To assess the impact of altered glycolytic metabolism on key parameters associated with cancer metastasis, we conducted cell migration assays (wound healing) and cell viability assays (MTT). As expected, TAM-R cells exhibited significantly faster migration than TAM-S cells, with migration in TAM-R cells reaching approximately 160% of the TAM-S cell migration rate (Figure 3A,B). Interestingly, when genistein was added to TAM-R cells, migration was significantly reduced and became comparable to the migration observed in TAM-S cells. This suggests that genistein may help inhibit metastasis in tamoxifen-resistant cells. Additionally, treatment with genistein significantly reduced cell viability in TAM-R cells at all concentrations tested, whereas no such effect was observed in TAM-S cells (Figure 3B).

### 2.5. Role of miR-155 Overexpression in Tamoxifen Sensitivity and Response to Genistein

**Figure 4:**
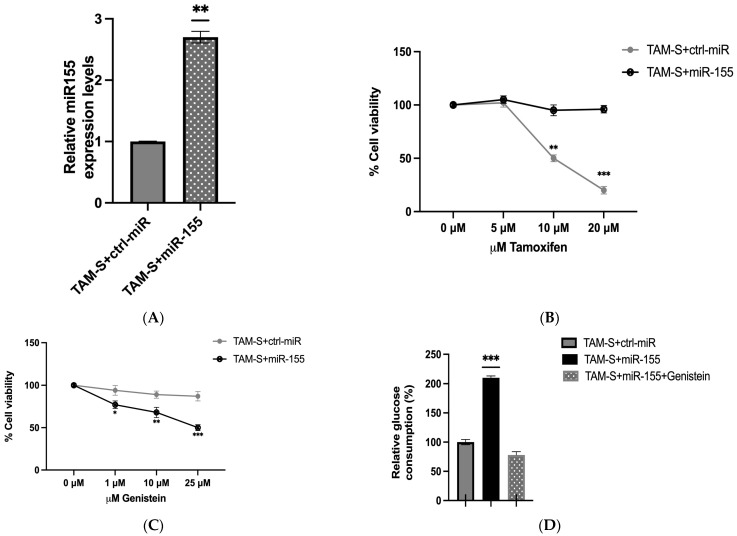
Effects of miR-155 overexpression on cell viability, glucose consumption and response to tamoxifen and genistein in TAM-S cells. (**A**) Expression levels of miR-155 in TAM-S cells expressing either control (ctrl)- TAM-S ctrl or miR-155 -TAM-S +miR155. (**B**) Effect of tamoxifen on cell viability of TAM-S control (ctrl-miR) and TAM-S expressing miR-155 (TAM-S + miR-155). (**C**) Effect of genistein (0–25 μM) on cancer cell viability in TAM-S ctrl-miR and TAM-S expressing miR-155 (TAM-S + miR-155), as measured by the MTT assay. (**D**) Relative glucose consumption in TAM-S control (ctrl-miR) and TAM-S expressing miR-155 (TAM-S + miR-155). Data are presented as mean ± SEM (*n* = 3). Statistical significance was set at *p* < 0.05. Significant differences relative to the control are indicated by asterisks: * *p* < 0.05, ** *p* < 0.01, *** *p* < 0.001.

Lastly, we investigated the impact of miR-155 overexpression on tamoxifen sensitivity in TAM-S cells. miR-155 was overexpressed in TAM-S cells to levels comparable to those observed in TAM-R cells (Figure 1B and Figure 4A). Interestingly, this resulted in a marked increase in resistance to tamoxifen treatment. Cell viability assays in TAM-S cells ectopically expressing miR-155 (TAM-S + miR-155) showed less than 5% reduction in cell viability following tamoxifen treatment, indicating a shift toward a resistant phenotype. TAM-S + ctrl-miR cells continued to respond to tamoxifen treatment as previously observed (Figure 1A and Figure 4B). These findings suggest that miR-155 plays a critical role in mediating tamoxifen resistance. Notably, treatment with genistein significantly reduced cell proliferation in cells overexpressing miR-155, suggesting that genistein may overcome the resistance conferred by elevated miR-155 levels (Figure 1C). Moreover, glucose consumption, a hallmark of the Warburg effect, was substantially reduced following genistein treatment, further supporting its role in modulating the Warburg effect in tamoxifen-resistant cells (Figure 4D).

## 3. Discussion

Tamoxifen resistance remains a significant challenge in the treatment of estrogen receptor-positive ER+ breast cancer. Our study provides valuable insights into the role of miR-155 in mediating tamoxifen resistance and highlights the potential therapeutic benefit of genistein, a natural isoflavone with known anticancer properties, as an adjunctive treatment for tamoxifen-resistant (TAM-R) breast cancer. We demonstrate that miR-155 is significantly upregulated in TAM-R breast cancer cells compared to tamoxifen-sensitive (TAM-S) cells, which is consistent with previous findings linking elevated miR-155 levels to poor prognosis and cancer progression in ER+ breast cancer [9]. These results suggest that miR-155 may play a crucial role in the molecular mechanisms driving tamoxifen resistance, potentially through the modulation of key signaling pathways, such as the STAT pathway. The upregulation of miR-155 in tamoxifen-resistant breast cancer also suggests its potential as a biomarker for predicting tamoxifen response.

Importantly, our data show that genistein significantly downregulated miR-155 expression in TAM-R cells, restoring sensitivity to tamoxifen. Specifically, at a concentration of 25 μM, genistein reduced miR-155 levels by more than three-fold, consistent with earlier reports demonstrating its ability to modulate miRNA expression in various cancer types [22,23,41]. Additionally, genistein treatment led to a significant reduction in HK2 expression, an enzyme central to the Warburg effect and cancer cell survival in aggressive tumors. This metabolic reprogramming suggests a potential link between genistein’s ability to combat tamoxifen resistance and its modulation of key metabolic pathways often altered in resistant cancer cells. Moreover, genistein treatment inhibited cell migration and proliferation in tamoxifen-resistant (TAM-R) cells, addressing the increased invasiveness typically observed in tamoxifen-resistant breast cancer. These findings highlight genistein’s potential to overcome tamoxifen resistance by targeting miRNA expression, particularly miR-155, and modulating crucial metabolic processes involved in tumor aggressiveness and drug resistance.

Genistein’s ability to downregulate miR-155 and modulate metabolic pathways may involve multiple mechanisms, including epigenetic regulation and direct interaction with transcription factors. Recent studies have highlighted several positive outcomes. A study in Sprague Dawley rats demonstrated that lifetime or adult genistein intake improved tamoxifen responsiveness and reduced the risk of recurrence This effect was linked to a reduction in the unfolded protein response, decreased autophagy and enhanced anti-tumor immune responses [42]. In vitro studies with BT-474 human breast cancer cells also revealed a synergistic inhibition of cell growth when combining tamoxifen and genistein. This synergistic effect was attributed to combined cell cycle arrest at the G1 phase, apoptosis induction and downregulation of key proteins, such as survivin, EGFR, HER2 and ERα [43].

Our findings that miR-155 is upregulated in tamoxifen-resistant (TAM-R) cells and may contribute to drug resistance highlight its potential as both a biomarker and a therapeutic target. Indeed, miR-155 has been proposed as a strong candidate for use as a biomarker [44]. In therapy, miR-155 could be used to predict patient responses to tamoxifen, enabling more personalized treatment strategies. For some patients, tamoxifen alone may be sufficient, while others could benefit from additional therapies. From a clinical trial perspective, genistein, with its potential for combination with existing therapies like tamoxifen, emerges as an attractive candidate for ongoing clinical testing [45].

While our in vitro findings are promising, it is important to note that the complex tumor microenvironment in vivo may influence the efficacy of genistein. Comprehensive in vivo experiments are needed to validate these results in more physiologically relevant contexts and elucidate the precise molecular mechanisms through which genistein exerts its effects. Exploring the interactions between miR-155 and other oncogenic pathways affected by genistein, as well as its potential synergistic effects with tamoxifen in preclinical models, remains critical. Genistein has shown promising potential in breast cancer treatment, particularly when combined with tamoxifen. Despite these promising findings, some studies have reported conflicting results, highlighting the complex interplay between genistein, breast cancer cells and tamoxifen. For example, a study demonstrated that low doses of dietary genistein (250 and 500 ppm) negated the inhibitory effect of tamoxifen on estrogen-induced MCF-7 tumor growth in ovariectomized athymic mice [46]. While these studies suggest caution in combining genistein with tamoxifen, they also highlight the need for further investigation to clarify the potential interactions between these compounds in breast cancer treatment.

Overall, the present findings highlight the critical need for continued research to fully elucidate the potential benefits and optimal use of genistein in combination with tamoxifen for breast cancer treatment. Although genistein shows promise as an adjunctive therapy, more comprehensive studies are required to confirm its efficacy and safety in clinical settings. Future research should focus on elucidating the precise molecular mechanisms of genistein’s action, its interactions with various breast cancer subtypes and its potential to enhance treatment outcomes in patients with tamoxifen-resistant ER+ breast cancer.

## 4. Materials and Methods

### 4.1. Cell Culture

Cell lines TAM-R and TAM-S MCF7 were generously provided by Dr. Robert Schneider from the NYU School of Medicine [28]. The cells were maintained in improved MEM (IMEM) supplemented with L-glutamine, 5% fetal bovine serum (FBS), 0.4% gentamicin sulfate, 0.5 μg/mL fungizone and 5 μg/mL plasmocin. Cultures were incubated at 37 °C in a 5% CO2 tissue culture incubator. To maintain the tamoxifen-resistant model, 1 μM of 4-hydroxytamoxifen (4-OHT) was added to the TAM-R cells.

### 4.2. Analysis of MIR155HG Expression in Breast Cancer Using KM-Plotter Database

The data for this study were retrieved from the KM-Plotter database (https://kmplot.com/analysis/, accessed on 13 December 2024), an online tool that integrates gene expression data from public datasets, including the Gene Expression Omnibus (GEO), European Genome-phenome Archive (EGA) and The Cancer Genome Atlas (TCGA) [29]. We conducted a Kaplan–Meier analysis of relapse-free survival in ER-positive breast cancer patients treated with endocrine therapy, focusing on MIR155HG expression. Specifically, data were obtained from the Kaplan–Meier analysis of relapse-free survival in ER-positive breast cancer patients treated with endocrine therapy. The analysis included 835 breast cancer patients, stratified into high-expression (*n* = 479) and low-expression (*n* = 356) groups based on MIR155HG expression levels. The optimal cutoff for group stratification was automatically determined by the KM-Plotter tool to maximize the separation between high- and low-expression groups. The statistical significance between these groups was assessed using the log-rank test, with a *p*-value threshold of <0.05 considered statistically significant. Hazard ratios (HRs) with 95% confidence intervals were calculated to quantify the association between MIR155HG expression and relapse-free survival.

### 4.3. Cell Viability/Proliferation Assays

The 3-(4,5-dimethylthiazol-2-yl)-2,5-diphenyltetrazolium bromide (MTT) assay was performed using the CellTiter 96 Non-Radioactive Cell Proliferation Assay according to the manufacturer’s instructions (Promega, Madison, WI, USA). Briefly, TAM-S and TAM-R MCF-7 cells were treated with tamoxifen (0–20 μM) or genistein (0–25 μM) for 24 h. Next, 150 mL/well of MTT [3-(4,5-dimethyl thiazol-2-yl)-2,5-diphenyl tetrazolium bromide] reagent was added, and the cells were incubated at 37 °C for 4 h, prior to measuring absorbance at 570 nm using a 96-well plate reader.

### 4.4. RT-qPCR

MicroRNA extraction was performed using the miRNeasy Mini Kit (Qiagen, Valencia, CA). MiRNA expression was quantified with the TaqMan miRNA RT-qPCR method (Applied Biosystems, Houston, TX, USA) on a CFX96 real-time PCR detection system (Bio-Rad, Hercules, CA, USA). For reverse transcription, 10 ng of total RNA was used, along with appropriate negative controls, including no-template and no-reverse-transcription controls, to ensure assay specificity. The primers and probes for Hsa-miR-155 and U6snRNA were sourced from Applied Biosystems and used according to the manufacturer’s guidelines. U6 snRNA served as an internal control for normalization due to its stable expression across samples. Relative expression levels were calculated using the 2^−ΔΔCt^ method. Overexpression of miR-155: Tamoxifen-resistant (TAM-R) MCF-7 cells were infected with control or miR-155 lentiviral vectors following the manufacturer’s instructions (Biosettia, CA, USA). Cell lines were selected based on their resistance to puromycin.

### 4.5. Immunoblot Analysis and Antibodies

Cell lysates were prepared and analyzed using standard immunoblotting techniques. Total protein was extracted, clarified via centrifugation at 4 °C, and protein concentration was determined using the Pierce BCA Protein Assay Kit (Thermo Fisher Scientific, Waltham, MA, USA). Equal amounts of denatured protein lysates were resolved by SDS-PAGE, transferred to a polyvinylidene difluoride (PVDF) membrane and blocked with 5% bovine serum albumin (BSA) for 1 h. Membranes were incubated overnight at 4 °C with primary antibodies at a 1:1000 dilution, targeting HK2 (Cell Signaling Technology, Catalog #2867), STAT3 (Catalog #9139), pSTAT3 (Catalog #9145) and β-tubulin (Catalog #2146).

### 4.6. Cell Migration/Wound Healing Assay

TAM-R and TAM-S MCF-7 cells (1.75 × 10^5^) were seeded in 6-well plates. At 90% confluency, wounds were created using a 200 µL pipette tip and washed with PBS. The base medium was improved MEM (Richter’s formulation) with L-glutamine, supplemented with 1% fetal bovine serum (FBS) and gentamicin, along with 25 µM genistein treatment as applicable. Images were captured at 0 and 24 h using the Invitrogen EVOS M5000 Imaging System from three experiments and were analyzed for percentage cell-covered area. Wound closure was analyzed as the ratio of the remaining wound area relative to the initial wound area [47].

### 4.7. Glucose Consumption

TAM-R and TAM-S MCF-7 cells were seeded onto a 6-well plate at a density of 3 × 10^5^ cells/well, and the culture medium was replaced with low glucose (Gibco; Thermo Fisher Scientific, Inc.). After 24 h of incubation, the glucose concentration within the cells was measured using a Glucose Assay Kit (Cell Biolabs, Inc., San Diego, CA, USA) according to the manufacturer’s instructions. Briefly, the cells were lysed, and viable cell numbers were determined using a cell viability assay (Trypan Blue exclusion). The lysate was then added to a 96-well microtiter plate. The reaction was initiated by adding the reaction mix, and the plate was incubated for 30 min at 37 °C, protected from light. Absorbance was measured at 560 nm using a microplate reader, and glucose concentration was quantified using a standard curve. Glucose consumption data were normalized to the viable cell count to account for potential differences in cell density and viability between conditions. Controls, including wells without cells to measure background glucose levels, were included to ensure accuracy.

### 4.8. Statistical Analysis

Data are expressed as the mean ± standard error of the mean (SEM) for a minimum of three biological replicates. All experiments were performed with both technical and biological triplicates (N = 3). Statistical analyses were conducted using GraphPad Prism^®^ version 10.3 (GraphPad Software, San Diego, CA, USA) and Microsoft Excel. The differences between two groups were assessed using a two-tailed Student’s *t*-test, while comparisons involving more than two groups were analyzed using one-way analysis of variance (ANOVA) with Tukey’s multiple comparison test. Statistical significance was defined as *p* < 0.05, with levels of significance indicated as * *p* < 0.05, ** *p* < 0.01 and *** *p* < 0.001. A 95% confidence interval (CI) was calculated for all estimates, and significant differences relative to the control group were marked with asterisks.

## Data Availability

Data are contained within the article.
